# A Reliable and Efficient Tracking System Based on Deep Learning for Monitoring the Spread of COVID-19 in Closed Areas

**DOI:** 10.3390/ijerph182412941

**Published:** 2021-12-08

**Authors:** Radwa Ahmed Osman, Sherine Nagy Saleh, Yasmine N. M. Saleh, Mazen Nabil Elagamy

**Affiliations:** 1 Basic and Applied Science Department, College of Engineering and Technology, Arab Academy for Science and Technology (AAST), Alexandria 1029, Egypt; 2 Computer Engineering Department, College of Engineering and Technology, Arab Academy for Science and Technology (AAST), Alexandria 1029, Egypt; mazenelagamy@aast.edu; 3 Computer Science Department, College of Computing and Information Technology, Arab Academy for Science and Technology (AAST), Alexandria 1029, Egypt; yasmine_nagi@aast.edu

**Keywords:** Internet of Things, Radio Frequency Identifier, deep learning, pandemic, COVID-19, Lagrange optimization, reliability, efficiency

## Abstract

Since 2020, the world is still facing a global economic and health crisis due to the COVID-19 pandemic. One approach to fighting this global crisis is to track COVID-19 cases by wireless technologies, which requires receiving reliable, efficient, and accurate data. Consequently, this article proposes a model based on Lagrange optimization and a distributed deep learning model to assure that all required data for tracking any suspected COVID-19 patient is received efficiently and reliably. Finding the optimum location of the Radio Frequency Identifier (RFID) reader relevant to the base station results in the reliable transmission of data. The proposed deep learning model, developed using the one-dimensional convolutional neural network and a fully connected network, resulted in lower mean absolute squared errors when compared to state-of-the-art regression benchmarks. The proposed model based on Lagrange optimization and deep learning algorithms is evaluated when changing different network parameters, such as requiring signal-to-interference-plus-noise-ratio, reader transmission power, and the required system quality-of-service. The analysis of the obtained results, which indicates the appropriate transmission distance between an RFID reader and a base station, shows the effectiveness and the accuracy of the proposed approach, which leads to an easy and efficient tracking system.

## 1. Introduction

There is no doubt that the world has been facing one of its biggest challenges since the World Health Organization (WHO) declared coronavirus disease (COVID-19) as an epidemic in December 2019 [1]. The consequences of this epidemic did not affect only the health domain, but also many areas such as the economy, education, and technology [2]. COVID-19 is caused by a coronavirus known as severe acute respiratory syndrome two (SARS-CoV-2), which was first identified in Wuhan, China [1,3,4]. It is not a new disease, but it is the evolution of an older virus known as SARS-CoV that was also discovered in China in 2003 [1,5]. 

Commonly, COVID-19 begins with mild symptoms that match those of influenza/bacterial pneumonia, such as cough and fever, then progressively can lead to death in severe cases [1,6,7]. Even though the vaccine for COVID-19 is now available, vaccinated people can still get infected. People in crowded areas, such as shopping malls, can easily spread this virus. The WHO declared that the best ways to prevent infection during the COVID-19 outbreak are still non-pharmaceutical measures such as social distancing, personal hygiene, disinfection of surfaces, and wearing masks and gloves [1,7,8,9]. This created a need to find efficient ways to identify suspected cases in crowded areas, which motivated many researchers to deploy the application of the Internet of Things (IoT) in physical distance monitoring and physical conditions tracking. 

Consequently, [10] developed an IoT investigation system, which supported identifying undocumented patients who showed no apparent symptoms and infectious places as well. In addition, their system allowed the identification of people who has close contact with an infected or suspected patient [10]. In September 2020, [11] proposed a privacy anonymous IoT model using RFID proof-of-concept, which granted mobile objects the ability to send/receive alerts when getting near a flagged, confirmed/expected infected case or flagged object/place. In addition, their model supported the identification of infection clusters’ contacts and distributed an alert for isolation purposes while conserving patients’ privacy [11]. The challenges and technical needs to deploy IoT and 5G-related technologies to support the prevention of COVID-19 spreading through offering novel solutions for contact tracing and telehealth were discussed in [12]. 

In October 2020, [13] developed a COVID-SAFE IoT framework, which aided in avoiding the spread of coronavirus. The COVID-SAFE framework was based on three main units: mobile application, IoT node, and fog-based Machine Learning (ML) tools. The IoT node was responsible for health conditions’ tracking, such as cough and respiratory rates, body temperature, and blood oxygen saturation, which was displayed using a mobile application and alerted the user to keep a safe physical distance of 2 m to control the spread of the virus. To predict virus spreading risk, the authors used a Fuzzy Mamdani real-time predictive system at the fog server and deployed two alternatives for the IoT node and fog server communication: LoRa or 4G/5G/WiFi [13]. 

Lately, in March 2021, [14] presented an IoT-based paradigm entitled IoT Based Paradigm for Medical Equipment Management Systems (IoT MEMS) to efficiently operate medical equipment in Intensive Care Units (ICUs). To provide fairness and transparency in allocating medical equipment they applied IoT technology to enhance the information flow between medical equipment management systems and ICUs during the COVID-19 pandemic [14]. 

To detect exposed places and prevent the spread of COVID-19, ref. [7] presented an ML approach for auditing COVID-19 infection risk measurements in public places using features that were extracted from IoT sensors then feed as the input for several ML algorithms, such as decision tree, random forest, support vector machine, neural network and naïve Bayes classifier, to calculate the risk probability and predict the risks of the COVID-19 infection. In August 2021, ref. [15] explained the benefits of using IoT, Artificial Intelligence (AI), Robotics, and Blockchain technologies in controlling the spread of COVID-19 and presented multidisciplinary techniques and applications such as Remote Patient Monitoring (RPM) by Wearable IoT (WIoT), tracing and tracking, Personal Digital Twins (PDTs), and risk prediction to encounter COVID-19 [15].

When collecting information from visitors in any closed area, there is an emergent need to transmit such data in a reliable and secure system to avoid loss or damage. Although various research has been proposed for tracking COVID-19 cases, the topic is still in need of further investigations in terms of gathering information to track possibly infected people and whether the collected data is reliable and accurate or not. Consequently, an efficient tracking system based on deep learning is proposed. The main goal of the proposed model is to assure that the received signal is accurate and efficient to facilitate tracking and increase the contagion control. The contributions of this article are summarized as follows:

- An efficient, reliable, and secure method is developed for transmitting suspected COVID-19 infected identification information through the proposed approach.
- An analytical model was formulated using an optimization problem to ascertain the reliability, efficiency, and security of a suspected COVID-19 infected identification information transmission.
- Based on the proposed approach, an efficient and reliable transmission system is designed using a one-dimensional convolutional neural network (1D-CNN) deep learning model, to predict the suitable transmission distance between an RFID reader and base station (BS) accurately.
- The proposed approach aims to enhance the transmission performance of the RFID sensor, which carries COVID-19 information. This is achieved through determining the optimum required transmission distance RFID reader and BS where the data will be stored.
- The proposed deep learning model is compared to state-of-the-art benchmark methods and provides a marked improvement in results.
- The proposed approach is investigated in terms of overall achievable data rate under different conditions, such as path loss exponent, RFID transmission power, interference transmission power, and different signal-to-interference-plus-noise ratio (*SINR_th_*) values. Based on these parameters, the whole network can be optimized in different environmental conditions.

The remainder of the article is organized as follows: In Section 2, the materials and methods will be presented. In Section 3, the experimental results will be reported. The discussion of all the results and limitations will be presented in Section 4. Finally, the paper will be concluded in Section 5. 

## 2. Materials and Methods

Using wireless technologies, such as IoT and fifth-generation (5G), the number of infected and uninfected people can be easily estimated and besieged [12]. Such technologies can be used to predict suspected COVID-19 cases, infected areas, and the percentage of virus spread [15,16]. Different types of wearable sensors [17] are commonly used to detect whether or not people are suspected of being infected with the virus based on signs such as temperature, blood oxygen, and coughing patterns [11,15]. Cameras, IoT sensors, and RFID sensors can track whether or not people are wearing masks and bound to a safe distance to avoid the infection, especially in closed areas such as malls, schools, universities, companies, and hotels [7,11]. 

In this section, the proposed idea is first presented, followed by a discussion of the security and privacy measures taken to ensure the reduction of possible vulnerabilities in the proposed system. The mathematical derivation representing the system model and problem formulation is then explained. Following this, a proposed deep learning model is presented, which learns from the simulation data and outputs an optimal distance to send data between an RFID reader and a base station. The data generation simulation will then be presented, followed by an analysis of the presented deep learning model.

### 2.1. Proposed Tracking System

**Figure 1:**
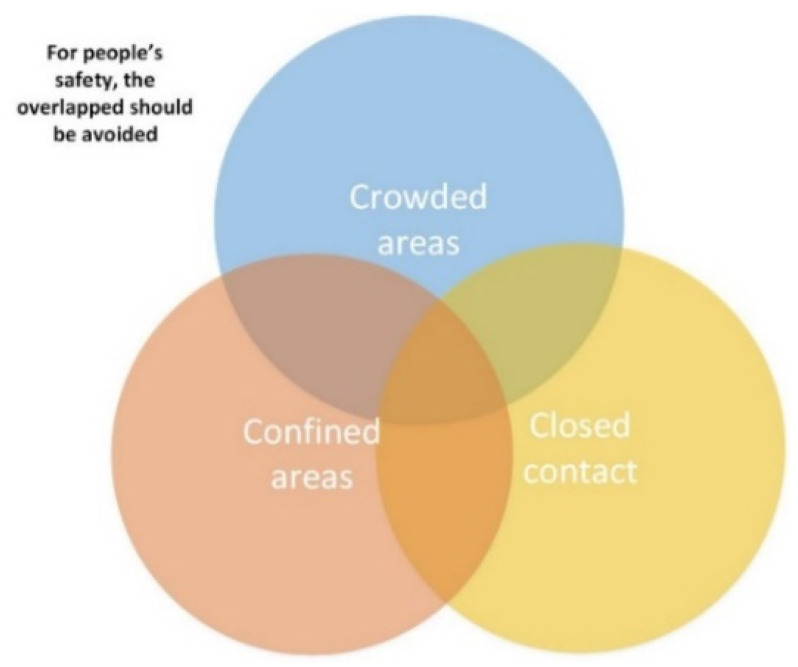
COVID safety precaution.

The proposed model aims to predict and track any COVID-19 patient to avoid or control infection spread. For safety considerations, people should avoid the intersection between crowded areas, confined spaces, and close contact with other people as shown in Figure 1. Inevitably, many people may not be able to avoid all these conditions at the same time and possibly get infected if they are not following the required precautions. The proposed model is designed to be deployed in any closed area such as malls, hotels, schools, hospitals and companies, where RFID is used to detect and track any COVID cases.

**Figure 2:**
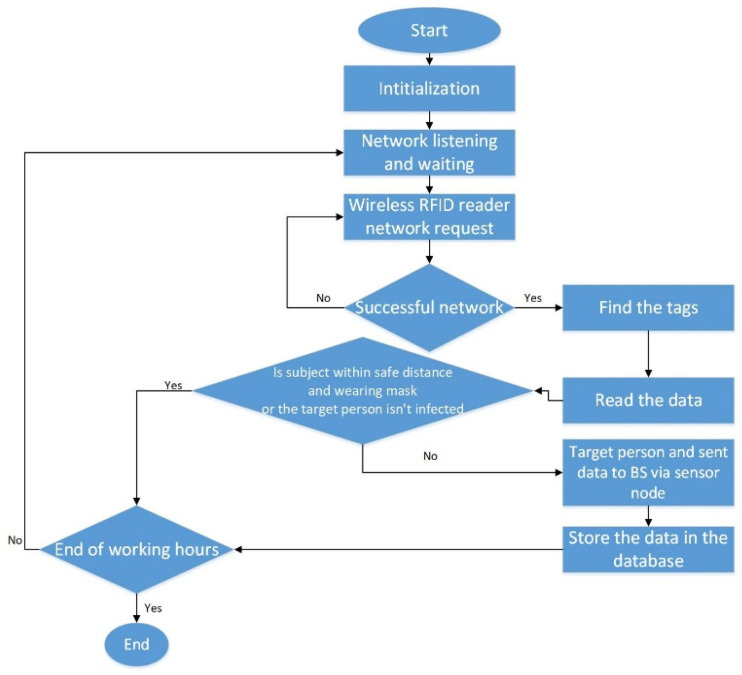
Proposed tracking system model flowchart.

For efficient and accurate tracking, it is assumed that people are wearing an RFID tag when entering any closed area, which will be connected to an RFID reader. The role of an RFID reader is to collect data required for COVID-19 detection continuously such as temperature, blood oxygen, and sensing the distance between any two people as shown in Figure 2. 

Two different scenarios are considered:

- If the data show a suspected infected person, the action will be taken immediately through the base station to request isolation of the target person and start tracking people who were in contact with him, if any;
- If the data show a situation that might indicate a possible virus spread, for example, a person who is not wearing a mask and the safety distance between him and any other person is less than the required COVID-19 safety distance, the data of this person should be stored in the database for at least one week more than the incubation period declared by the WHO until it is confirmed that there are no COVID-19 cases reported.

If any COVID-19 cases are reported, all the people who were in contact should be tracked and isolated until it is confirmed that they are safe. The following probabilities are considered to help detect those to be tracked:

- P(A) is the probability that the person is not wearing a mask.
- P(B) is the probability that the distance is less than the defined safety distance.
- P(C) is the probability that at least one of the two persons has a fever.
- P(D) is the probability that at least one of the two persons has low blood oxygen.
- P(E) is the probability that two close persons are wearing masks.
- P(F) is the probability that the contact distance is more than the safety distance.

The model presented in the following subsections assures that the above probabilities are calculated based on reliable, efficient, and accurate data received from the RFID reader. Table 1 shows an example of the action to be recommended based on the different combinations of probabilities.

Table 1 is considered as a guide for the proposed model to decide which data should be sent and stored in the base station. At any closed area, a thousand people can be found; then, if all the information is sent to any base station it may cause network overhead or system failure. Therefore, the information that should be sent is only the information of the people who did not satisfy the safety conditions as explained in Table 1. Based on deciding which data should be sent, the proposed approach will be responsible for sending these data in an efficient, reliable, and secure way to easily track any reported infected person in time.

### 2.2. Security and Privacy Measures 

Multiple studies have been conducted to investigate the security and privacy vulnerabilities occurring due to the deployment of RFIDs in diverse applications, especially those involving the processing of personal information as in [18,19,20,21]. According to [20], the use of RFID tags for tracking can suffer from diverse security and privacy attacks such as fabrication, interception, modification, domination and interruption. 

Fabrication attacks on RFIDs include attacks on entities (such as RFID tag switching, RFID cloning, location attacks, and social engineering attacks) and attacks on packets (replay attacks) [20]. In the proposed Covid-19 tracking system, fabrication attacks could be conducted as follows: Since the information stored on RFID tags could be read from an existing tag and cloned to a blank tag, RFIDs could suffer from tag cloning. A malicious person could clone an RFID tag or even switch it with an old one, which could result in the wrong identification and tracking of potential virus carriers inside closed areas. RFID cloning could be suppressed by the deployment of security chips and cryptographic functions or physical unclonable functions [21]. Attacks on packets such as replay attacks are a result of replaying old messages. Attackers could replay old messages to gain access to previously visited places that might have access restrictions due to a limitation on the number of admitted people for social distancing purposes. Replay attacks could be thwarted by using authentication protocols to ensure data freshness [20].

Interception attacks on RFIDs include eavesdropping and object tracking [20]. Eavesdropping could allow attackers to learn sensitive information about people being tracked in closed areas. Consequently, personal information revealing the identity and contact information of the people wearing the RFID tags are not stored on the tags. Sensitive information, such as personal identification details related to every RFID tag, is encrypted and stored in data centers or BS and is only accessed by authorized trusted personnel when needed. This personal information will only be stored for at least one week more than the incubation period announced by the WHO.

Modification attacks might be conducted when an adversary modifies the function or protocol of RFID readers [20]. This could prohibit readers from efficiently identifying tags in their vicinity thus leading to unreliable tracking. One way to address this attack is to use image processing algorithms to study camera feeds from video cameras covering the same areas of the readers and compare the number of reported people with that identified by the readers. In addition, since standard communication protocols are deployed for the RFID tag readers, an RFID tag tracking security threat could result when an adversary uses his/her reader to collect information from the nearby tags without the consent of the tag holders [20,21]. Consequently, the security and privacy of the tag holder could be compromised as his/her tag could be tracked at different locations in the closed area. In [21], several approaches have been suggested for the protection against tag tracking such as probabilistic encryption and the use of hash chains.

Domination attacks on RFID tags could target the cracking of the keys between the tags and the readers, which may affect the efficiency of tracking inside closed areas. This could be mitigated by the constant change of the keys or by the use of long keys [20]. Finally, interruption attacks include Denial of Service (DoS) attacks. In general, DoS attacks could be defined as a strive to cease network services and resources for legitimate users in computer systems or networks, which affect availability and reliability. This happens when a device or server is under attack due to intentional false requests generated by an attacker to flood the communication channel and consume all available bandwidth, which prevents legitimate users from acquiring the requested services [22,23]. 

When DoS attacks are conducted from different sources, this is considered a Distributed Denial of Service (DDoS) [23]. DDoS attacks target diverse organizations (ranging from private to public governmental entities such as health and education) and could have serious long-term damaging implications for businesses such as compromising reputation, financial losses, the addition of operational costs, and possible loss of customers [22,23]. In RFID-based systems, DoS attacks could be carried out by applying noise interference to jam the systems, block radio signals or tamper with the RFID tag (disabling the tag or modifying the RFID tag data) [20,24]. 

Several mitigation techniques have been developed to deal with DoS attacks such as the deployment of strong authentication mechanisms, the use of physical unclonable functions (PUF) [25], the possible use of alarms triggered when a tag is being tampered with, and the constant update of the RFID devices [20]. In [26], a comparative study has been presented, which studies different RFID authentication protocols and highlights those protocols which handle DoS attacks. As for the battery drainage due to DoS attacks, strong authentication protocols will be deployed in the proposed system to try to reduce the possibility of these attacks to the minimum. In addition, since this system is proposed for an enclosed area, constant power supplies could be available to ensure that, even if batteries are depleted, they can be easily recharged to keep the readers running. 

### 2.3. Analytical Model

The proposed analytical model assumes that there are *N* people in a closed area such as a mall, shop, company, each of them is going to wear an RFID tag. An RFID reader is going to continuously read the temperature, distance between any two users, if he is wearing a mask or not. The number of users (*N*) that each RFID reader can read their information can be calculated as follows:

(1) $$N={\left[\frac{d_{TR}}{d_{safe}}\right]}^{2}*\;4,$$

where $d_{TR}$, $d_{safe}$ are the default distances between RFID tag and RFID reader respectively [27]. According to the WHO, the safety distance between any two users is considered to be at least 1 m [28]. On the other hand, the centers for disease control (CDC) considers the safety distance to be 6 feet (approximately 1.8 m) [9]. Accordingly, in the proposed model, the safety distance is assumed to be 2 m. The distance between any RFID tag and RFID reader can be expressed as [27]:

(2) $$d_{TR}=\sqrt{\frac{P_{R}\;G_{tag}\;G_{reader}\;\tau }{P_{T}}}*\frac{\lambda }{4\pi }$$

where *P_R_* is the reader transmission power received, *P_T_* is the tag transmitted power. *λ* is the wavelength of the Radio Frequency signal. Symbols *G_tag_* and *G_reader_* are the gain of tag-antenna and reader-antenna, respectively. τ is the transmission coefficient.

One of the important factors that should be addressed for the proposed approach is network scalability. Scalability is defined as the ability of the network to handle a huge number of users. In the proposed approach, there are multiple numbers of RFID readers, which are uniformly distributed, covering all indoor space, to ensure scalability of the network as the number of users increases and ensure that all RFID readers have the same transmission power. Each RFID reader serves around a thousand people. For the proposed approach, and based on Equation (1), it is assumed that each RFID reader will serve up to 1089 users. Therefore, as the number of closed-area visitors increases (reaching its peak especially during the holidays and special occasions), the densely distributed RFID readers will be able to handle the huge number of tags without affecting their performance efficiency. Additionally, in the proposed model, there are several base stations, each will be responsible for receiving data from some, not all, RFID readers. Furthermore, if the capacity increases and the number of RFID readers increases, then more base stations must be deployed.

For the proposed model, a Rayleigh fading channel with additive white Gaussian noise (AWGN) is considered. Additionally, the proposed model is subjected to a path loss and a statistically mutually independent fading channel coefficient for all transmission links.

The proposed model aims to receive the maximum number of the reliable and efficient required information to detect a possibility of an infected COVID-19 person and calculate the probability of increasing infection. This goal is achieved by finding the optimum required transmission distance between an RFID reader and BS under different environmental and channel conditions. Consequently, the equation that expresses the aims of the proposed model can be formulated as:

(3) $$Max\;\left\{R_{T}\right\}\;s.t.\;p_{out}\leq 1-U\;s.t.\;P_{R}\leq P_{Rmax},$$

where *R_T_* is the overall system achievable data rate in bit/s. Symbols $p_{out}$ and U represent the system outage probability and the required QoS, respectively. Parameters $P_{R}$ and $P_{Rmax}$ represent the RFID reader transmission power and the maximum RFID reader transmission, respectively. Symbols $R_{T}$ and $p_{out}$ can be expressed as [29]:

(4) $$R_{T}=B\;log_{2}\left(SNR_{TR}+SINR_{RBS}\right)$$

(5) $$p_{out}=p_{outTR}+p_{outRBS}-p_{outTR}*p_{outRBS},$$

where $SNR_{TR}$ and $SINR_{RBS}$ are the signal-to-noise ratio between RFID tag and RFID reader and the signal-to-interference-plus-noise ratio between an RFID reader and BS. Symbols $p_{outTR}$ and $p_{outRBS}$ are the transmission link outage probability between RFID tag and RFID reader and between an RFID reader and BS, respectively, which can be given as [29,30]:

(6) $$p_{outTR}=p\left(SNR_{TR}<\gamma_{th}\right)$$

(7) $$p_{outRBS}=p\left(SINR_{RBS}<\beta_{th}\right)$$

(8) $$SNR_{TR}=\frac{P_{T}{\left|h_{TR}\right|}^{2}}{\sigma^{2}}$$

(9) $$SINR_{RBS}=\frac{P_{R}{\left|h_{RBS}\right|}^{2}}{P_{I}{\left|h_{IBS}\right|}^{2}+\sigma^{2}}$$

(10) $$p_{outTR}=1-e^{-\gamma_{th}\left(\frac{\sigma^{2}}{P_{T}{\left|h_{TR}\right|}^{2}}\right)}$$

(11) $$p_{outRBS}=1-\frac{P_{R}{\left|h_{RBS}\right|}^{2}}{\beta_{th}\;P_{I}{\left|h_{IBS}\right|}^{2}+P_{R}{\left|h_{RBS}\right|}^{2}}\;e^{-\beta_{th}\left(\frac{\sigma^{2}}{P_{R}{\left|h_{RBS}\right|}^{2}}\right)},$$

where $\gamma_{th}$ and $\beta_{th}$ represent the threshold signal-to-noise ratio between RFID tag and RFID reader and the threshold signal-to-interference-plus-noise ratio between an RFID reader and BS. $P_{T}$ and $h_{TR}$ are the RFID tag transmission power and channel gain coefficient between RFID tag and RFID reader, respectively. $\sigma^{2}$ denotes the variance of the Additive White Gaussian Noise (AWGN) with zero mean. Parameters $h_{RBS}$ and $h_{IBS}$ are the channel gain coefficients between an RFID reader and BS; and between any interfere device and BS, respectively. Symbol $P_{I}$ is the interference transmission power. 

Assuming $\beta_{th}\;\sigma^{2}\ll P_{R}{\left|h_{RBS}\right|}^{2}$, then Equation (11) can be written as [31]:

(12) $$p_{outRBS}=1-\frac{P_{R}{\left|h_{RBS}\right|}^{2}}{\beta_{th}\;P_{I}{\left|h_{IBS}\right|}^{2}+P_{R}{\left|h_{RBS}\right|}^{2}}.$$

Thus, Equation (5) can be written as:

(13) $$p_{out}=1-\left(\frac{P_{R}{\left|h_{RBS}\right|}^{2}}{\beta_{th}\;P_{I}{\left|h_{IBS}\right|}^{2}+P_{R}{\left|h_{RBS}\right|}^{2}}e^{-\gamma_{th}\left(\frac{\sigma^{2}}{P_{T}{\left|h_{TR}\right|}^{2}}\right)}\right),$$

where ${\left|h_{TR}\right|}^{2}$ and ${\left|h_{RBS}\right|}^{2}$ can be expressed as [32,33]:

(14) $${\left|h_{TR}\right|}^{2}=\frac{{\left|h_{oTR}\right|}^{2}}{PL_{oTR}d_{TR}{}^{\alpha }}$$

(15) $${\left|h_{RBS}\right|}^{2}=\frac{{\left|h_{oRBS}\right|}^{2}}{PL_{oRBS}d_{RBS}{}^{\alpha }}$$

(16) $${\left|h_{IBS}\right|}^{2}=\frac{{\left|h_{oIBS}\right|}^{2}}{PL_{oIBS}d_{IBS}{}^{\alpha }},$$

where ${\left|h_{oTR}\right|}^{2}$, ${\left|h_{oRBS}\right|}^{2}$ and ${\left|h_{oIBS}\right|}^{2}$ follow a complex normal distribution CN~(0, 1). $PL_{oTR},$ $PL_{oRBS}$ and $PL_{oIBS}$ are the pathloss constant between RFID tag and RFID reader, between RFID reader and BS, and between any interfering device and BS, respectively. $d_{TR}$, $d_{RBS}$ and $d_{IBS}$ represent the distance between RFID tag and RFID reader, between RFID reader and BS, and between interfering transmission devices and BS, respectively. *α* is the pathloss exponent.

#### Problem Formulation

In this section, the optimization problem of the proposed model is analyzed. The main objective of the performance optimization of the proposed model is to maximize the overall system data rate under different environmental conditions. To find the solution of the optimization problem stated in Equation (3), the first-order optimality conditions can now be investigated. The Lagrangian of the optimization problem can be calculated as:

(17) $$l\;\left\{R_{T},\;\lambda ,\;\mu \right\}=R_{T}+\lambda \;\left(1-U-p_{out}\right)+\mu \;\left(P_{Rmax}-P_{R}\right).$$

where *λ* and *μ* are the non-negative Lagrangian multipliers, by taking the derivative of Equation (17) with respect to $d_{RBS}$ and $P_{R}$. Then, the optimal solution of Equation (3) can be solved as:

(18) $$\frac{\partial l\left\{R_{T},\;\lambda ,\;\mu \right\}}{\partial d_{RBS}}=0.$$

(19) $$\lambda =\left(\frac{{\left(P_{I\;}{\left|h_{IBS}\right|}^{2}\;\beta_{th}\;PL_{oRBS}\;d_{RBS}{}^{\alpha }+P_{R}\right)}^{2}\sigma^{2}*B}{\left(e^{-\gamma_{th}\left(\frac{\sigma^{2}}{P_{T}{\left|h_{TR}\right|}^{2}}\right)}\right)\;\left({\left(P_{I\;}{\left|h_{IBS}\right|}^{2}PL_{oRBS}\;d_{RBS}{}^{\alpha }+\sigma^{2}\right)}^{2}\left(\sigma^{2}+P_{T}{\left|h_{TR}\right|}^{2}\right)+P_{R\;}\sigma^{2}\;\left(P_{I\;}{\left|h_{IBS}\right|}^{2}PL_{oRBS}\;d_{RBS}{}^{\alpha }+\sigma^{2}\right)\right)}\right)$$

(20) $$\frac{\partial l\left\{R_{T},\;\lambda ,\;\mu \right\}}{\partial P_{R}}=0.$$

(21) $$\begin{array}{c} \mu =(\frac{\sigma^{2}*B}{P_{R}\sigma^{2}+\sigma^{2}(P_{I}{\left|h_{IBS}\right|}^{2}PL_{oRBS}d_{RBS}{}^{\alpha }+\sigma^{2})+P_{T}{\left|h_{TR}\right|}^{2}(P_{I}{\left|h_{IBS}\right|}^{2}PL_{oRBS}d_{RBS}{}^{\alpha }+\sigma^{2})})\\ -\lambda \frac{e^{-\gamma_{th}(\frac{\sigma^{2}}{P_{T}{\left|h_{TR}\right|}^{2}})}P_{I}{\left|h_{IBS}\right|}^{2}\beta_{th}PL_{oRBS}d_{RBS}{}^{\alpha }}{{\left(P_{I}{\left|h_{IBS}\right|}^{2}\beta_{th}PL_{oRBS}d_{RBS}{}^{-\alpha }+P_{R}\right)}^{2}} \end{array}$$

Equations (19) and (21) show the value of λ and *μ* that satisfy the constraint of the optimization problem. Next the derivative of Equation (17) with respect to λ and *μ*, then the optimal solution can be written as:

(22) $$\frac{\partial l\left\{R_{T},\;\lambda ,\;\mu \right\}}{\partial \lambda }=0.$$

(23) $$d_{RBS}={\left[\frac{e^{-\gamma_{th}\left(\frac{\sigma^{2}}{P_{T}{\left|h_{TR}\right|}^{2}}\right)}P_{R}-UP_{R}}{P_{I\;}{\left|h_{IBS}\right|}^{2}\;\beta_{th}\;PL_{oRBS}}\right]}^{\frac{1}{\alpha }}$$

(24) $$\frac{\partial l\left\{R_{T},\;\lambda ,\;\mu \right\}}{\partial \mu }=0.$$

(25) $$P_{R}=P_{Rmax}.$$

Symbols $d_{RBS}$ and $P_{R}$ represent the optimum required distance between an RFID reader and BS and the optimum required RFID transmission power, respectively, to receive reliable and efficient data. The obtained results of the proposed approach using a numerical and deep learning algorithm will be presented in Section 3.

### 2.4. Proposed Deep Learning Model

IoT systems are based on the presence of a vast number of sensors collecting information all the time. The collected information needs to be efficiently transmitted from each RFID reader to a base station for further processing. In real life, the transmission of such data can be affected by environmental factors, such as signal-to-noise ratio, or sensor-related parameters such as the power of transmission. Therefore, determination of the optimal distance according to which an RFID can send the data reliably to the base station is needed. Since the factors affecting transmission could change, the presence of a machine learning-based system helps to build an initial model that could then be updated whenever needed [34,35]. According to the analytical equations presented in Section 2.3, in the proposed system the variables U, $P_{R}$, *SINR_th_* and $d_{IBS}$ could affect the optimal distance $d_{RBS}$ of reliable data transmission between the RFID reader and the base station. 

In this subsection, a proposed deep learning model is presented that is intended to be used by the RFID readers to calculate the optimal distance to send the information regarding possible COVID-19 infected and people violating the minimum approved distance given different environmental factors that would affect the reliability and efficiency of the transmission. The proposed model was built using a 1D-CNN [36], which has recently been employed in several applications concerning signal processing [37] and 5G IoT interference avoidance [38].

The choice of using the 1D-CNN in the proposed model was based on multiple advantages that were discussed in previous research [36,37,38]. For instance, a 1D-CNN based model has low computational complexity which makes it easy to rapidly build and deploy. This also makes it suitable for use in real-time and for applications that have a limited power supply. Furthermore, the 1D-CNN based models have shown improved results when compared to traditional deep learning models [38]. 

**Figure 3:**
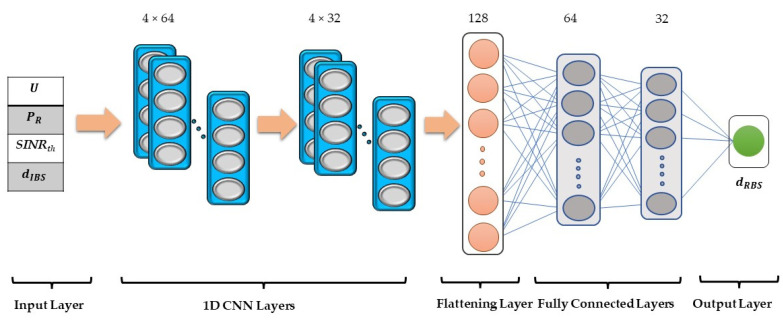
Proposed deep learning model.

A grid search was performed to find the optimum deep learning model for the proposed idea. The model was tested for different combinations of one or two or three 1D-CNN and fully connected hidden layers. Each 1D-CNN was experimented on with 32 or 64 or 128 filters having different kernel sizes and the fully connected layers for 32, 64, and 128 nodes. Furthermore, a different number of training epochs ranging between 50 and 250 were tested. The best results were achieved by the model presented in Figure 3 and is to be explained as follows:

Before the data are input to the deep learning model, it was scaled according to the min-max normalization presented by the equation:

(26) $$x_{new}=\frac{x-x_{min}}{x_{max}-x_{min}},$$

where $x_{new}$ represents the normalized value, x is the value to be normalized, $x_{min}$ and $x_{max}$ are the minimum and maximum values of x, respectively.

The proposed model starts with the four normalized variables that are to be collected, at the time an RFID reader intends to send information to the BS thus needs to calculate the optimal distance to send it efficiently and reliably. The input data represent the required QoS (U), transmission power ($P_{R}$), a threshold signal-to-interference-plus-noise ratio (*SINR_th_*) and distance between interfering transmitting devices and the base station $(d_{IBS}$). The data from the input layer are propagated through two hidden 1D CNNs. The first 1D-CNN has 64 filters, a kernel of size 4, and the output is padded thus reserving the matrix dimension to be input to the following 1D-CNN of 32 filters. 

The output from the 1D-CNN layers is then input to a flattening layer, therefore resizing it to suit the fully connected layers. The sequence of fully connected layers would predict the regression value of the optimum distance $\left(d_{RBS}\right)$ required to best transmit the information. The activation function adopted for all the hidden layers is the Rectified Linear Unit (ReLU). ReLU outputs the same input values except for negative ones, which are output as zero. Since the model is calculating a distance, the output value cannot be negative. For the output layer, Parametric ReLU (PReLU) was adopted since it has an extra parameter that is adaptively learned for negative values, thus finetuning the estimate for distances close to zero. The model was trained for 200 epochs while applying the adaptive moment estimation (Adam) optimization function [39], which adaptively optimizes the learning process while using the mean absolute error loss function as a target.

The assessment of the proposed deep learning model and comparison to other benchmarks were based on both the mean absolute error and the mean square error. The mean absolute error (MAE) and the mean squared errors are calculated as:

(27) $$\mathrm{MAE}=\frac{1}{n}\;\overset{n}{\underset{i=1}{\sum }}\left|y_{i}-{\hat{y}}_{i}\right|$$

(28) $$\mathrm{MSE}=\frac{1}{n}\;\overset{n}{\underset{i=1}{\sum }}{\left(y_{i}-{\hat{y}}_{i}\right)}^{2},$$

where n is the total number of records and $y_{i}-{\hat{y}}_{i}$ is the difference between the actual and the predicted values. 

### 2.5. MATLAB Simulation

According to the proposed deep learning model presented in the previous section, the parameters needed for training are U, $P_{R}$, *SINR_th_*, $d_{IBS}$, and $d_{RBS}$. MATLAB simulation was used to generate different values of the output distance using the optimized equations presented in Section 2.3 while applying the parameters described in Table 2. The dataset helps the deep learning model learn how to generate the optimal distance between an RFID reader and a BS, given different situations in real-time.

Table 3 shows the statistical description of all the input and output variables in the generated dataset. The range of the variables U, $P_{R}$, *SINR_th_* and $d_{IBS}$, as specified in Table 3, were used in the MATLAB simulation as inputs and, accordingly, the values of the variable ($d_{RBS}$) were generated for all records based on the analysis presented in Section 2.3. This resulted in a dataset of 90,288 unique records. Each record represented a different combination of values for the input variables and the equivalent optimum output distance $d_{RBS}$. The dataset generated is to be used by the initial deep learning model to learn to calculate the optimum $d_{RBS}$ that will improve the reliability and efficiency of the data transmission. 

**Figure 4:**
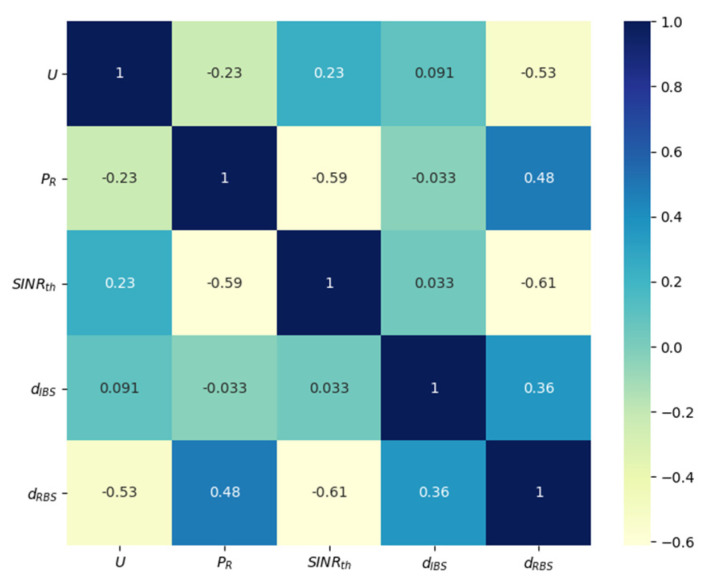
Pearson correlation of all variables.

In addition, Figure 4 shows the Pearson correlation of the variables. The figure shows that the distance to be calculated ($d_{RBS})$ is not highly correlated with any of the input variables thus making it challenging to predict with minimum error.

### 2.6. Deep Learning Model Assessment

The proposed deep learning model was assessed based on an average of 10-cross validation experiments, where the same splits were used for comparing the results of different benchmarks to assure a fair comparison. The data input to the benchmarks were also normalized using min-max normalization, similar to the proposed model. The benchmarks used were linear regression (LR), Adaboost regression (Ada), support vector regressor (SVR), and multilayer perceptron regressor (MLP). The Ada, SVR, and MLP were first tested independently in a grid search to learn optimal parameters for them, thus assuring that the comparison with the proposed model is fair. The optimal parameters generated for Ada were a learning rate of 0.1, exponential loss, and 150 estimators. For the SVR, the optimal parameters generated were a radial basis function kernel and gamma equal to 50. Finally, the MLP produced the best results using a ReLU activation, alpha of 0.0001, adaptive learning rate, and Adam optimizer.

The average of the MAE and MSE for the 10 folds results on each benchmark are represented in Table 4. The results show that the proposed 1D-CNN model outperforms all other benchmarks in terms of both the MAE and MSE, where the proposed model achieved the least MAE of 0.18 m and MSE of 0.09 on the testing data. 

**Figure 5:**
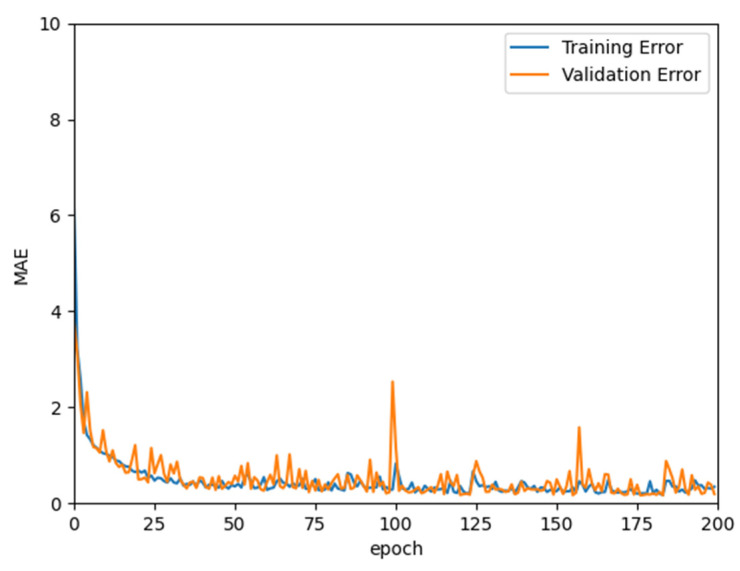
Mean absolute error generated by training and validation data.

Another experiment was performed to assess the proposed model using two-thirds training and one-third testing data splits. Figure 5 shows the mean absolute error resulting from both the training and validation datasets. The figure assumes that the model is not overfitted to the data and that the results hardly change after the 50th epoch, thus it was sufficient to stop the training at 200 epochs. The results obtained from the one-third testing will be used in the analytical results presented in the following section.

## 3. Results

**Figure 6:**
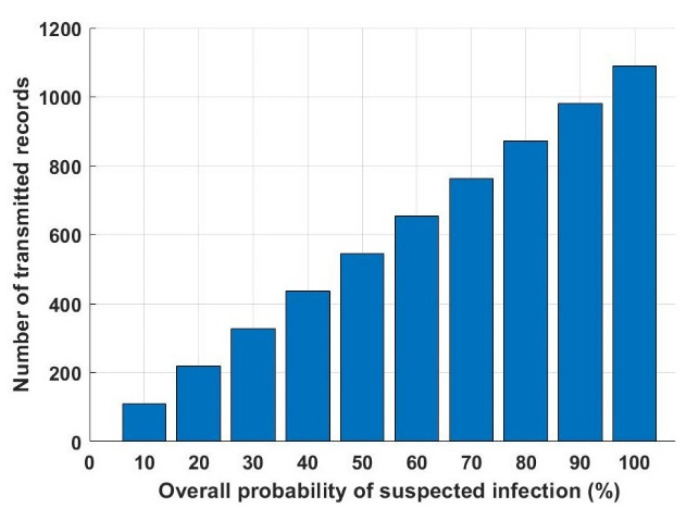
The overall probability of suspected infection versus the number of transmitted records.

**Figure 7:**
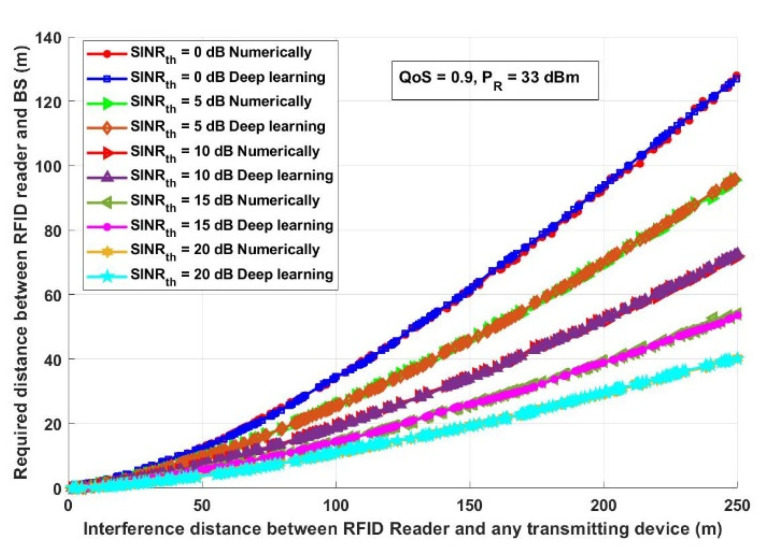
Interference distance between an RFID reader and any transmitting devices (m) versus required distance between RFID and BS (m).

**Figure 8:**
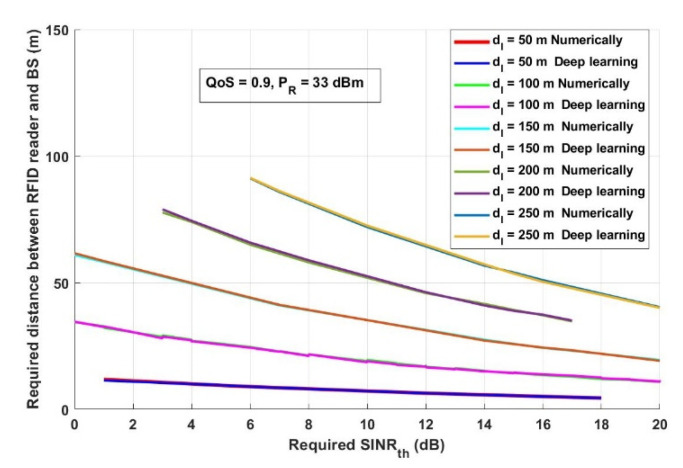
Required *SINR_th_* (dB) versus required distance between RFID and BS (m).

**Figure 9:**
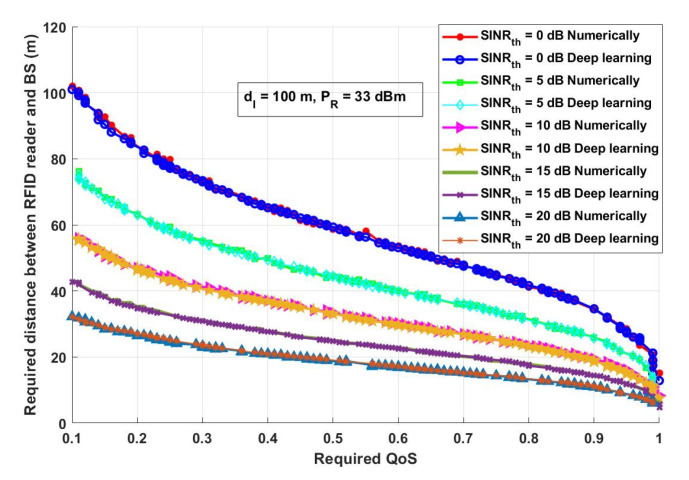
Required QoS versus required distance between an RFID reader and BS (m).

**Figure 10:**
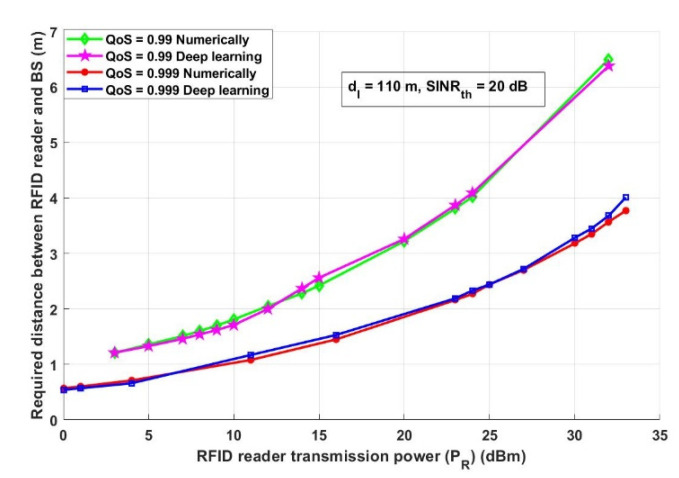
RFID reader transmission power (*P_R_*) (dBm) versus the required distance between an RFID reader and BS (m).

In this section, the results achieved by both the numerical analysis and the deep learning model are presented and thoroughly explained. The results obtained from the two-third training and one-third testing split experiment presented in Section 2.5 are compared with the analytical results obtained from the mathematical derivations presented in Section 2.3. Figure 6 depicts the overall probability of suspected infections versus the required transmitted records, which will be transmitted, saved, and tracked through the proposed model. Figure 7 shows the interference distance between an RFID reader and any transmitting device within a closed area (such as malls, schools, universities, companies, and hotels) versus the required distance between an RFID reader and BS. For the same network conditions stated in Figure 7, Figure 8 depicts the required distance between an RFID reader and a BS with different required system *SINR_th_*. The proposed approach is evaluated again, in Figure 9, in terms of the required distance between an RFID reader and a BS but versus the system-required QoS. Assuming that the *d_IBS_* is 100 m and the RFID reader transmission power (*P_R_*) is 33 dBm. The effect of an RFID reader transmission power on the system performance is shown in Figure 10.

**Figure 11:**
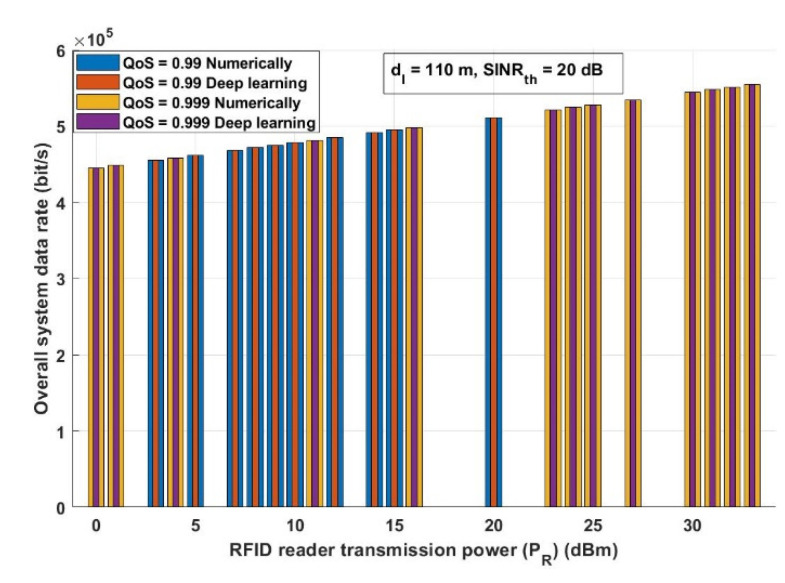
RFID reader transmission power (*P_R_*) (dBm) versus overall system data rate (bit/s).

**Figure 12:**
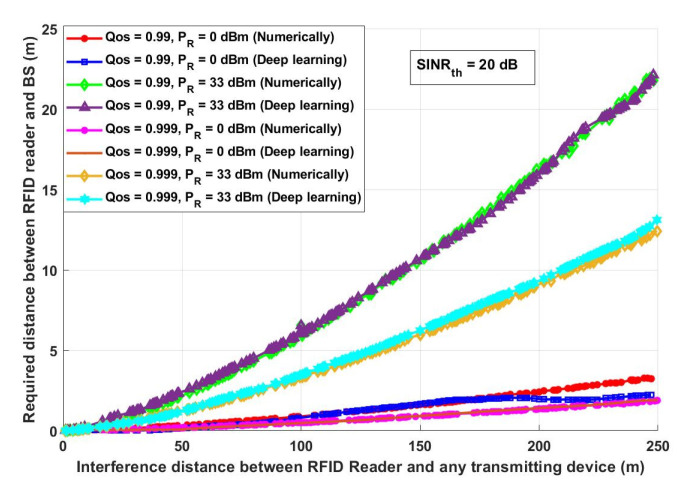
Interference Distance between an RFID reader and any transmitting devices (m) versus required distance between RFID and BS (m).

**Figure 13:**
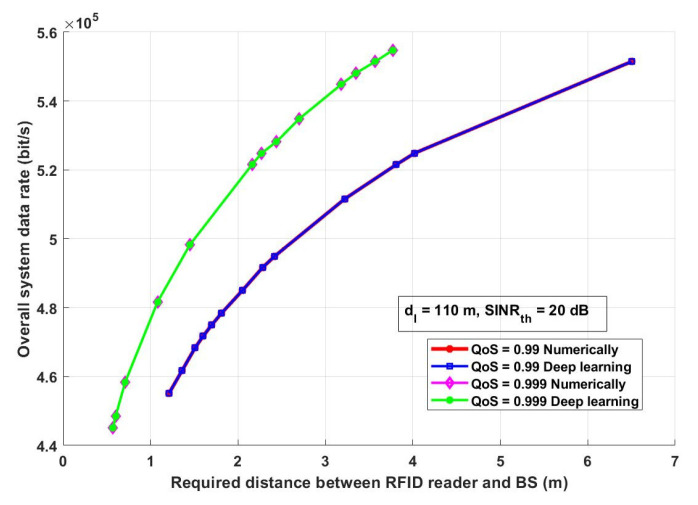
The required distance between an RFID reader and BS (m) versus overall system data rate (bit/s).

The overall system data rate is one of the parameters that should be investigated as it reflects the quality and the performance of the system. Assuming the same system parameters stated in Figure 10, Figure 11 shows the effect of the RFID transmission power (*P_R_*) on the overall system data rate. Figure 12 evaluates the presented COVID-19 tracking system from a different aspect. In this figure, it is assumed that there are two different QoS values: 0.99 and 0.999 with two different *P_R_*: lowest *P_R_* (0 dBm) and highest *P_R_* (33 dBm) and with *SINR_th_* equal to 20 dB. Figure 13 depicts how the proposed approach can enhance the system performance.

## 4. Discussion

For the proposed model, as mentioned in Section 2.3, there could be up to 1089 people detected by one RFID reader at a time. The number of the transmitted records depends on the percentage of possibly infected visitors based on the overall probability of satisfactory and unsatisfactory conditions. Figure 6 depicts the number of required transmitted records that should be sent, saved, and tracked. As shown in Figure 6, it is worth mentioning that the required saved data increases when the overall probability of suspected visitors in the area covered by an RFID reader not following the required safety precaution increases. For example, when 5% of visitors in an area did not satisfy the precaution conditions, then the reader will send around 55 records to be stored at the base station. However, if this percentage is increased to 20% then the reader has to send around 218 records to the base station for storing the data for future tracking if necessary. Additionally, if all the visitors did not satisfy the required precaution conditions, therefore 100% unsatisfactory conditions, then 1089 records should be sent and stored at the base station.

Assuming that the reader power is 33 dBm [27] and the required system QoS equals 0.9, it can be observed from Figure 7. That, to achieve the highest reliable and efficient data about any COVID-19 case, the RFID reader must be allocated at a specific distance. This distance is determined based on the network parameters, for example, when the *SINR_th_* is 0 dB along with an interference distance between an RFID reader and any transmitting device of 150 m, therefore the distance between an RFID reader and BS to obtain reliable information should be approximately 61.62 m. This was deduced using the analytical model and also calculated using the deep learning model. However, when *SINR_th_* is 20 dB along with an interference distance of 150 m, the distance between an RFID reader and BS to obtain reliable information should be approximately 19.5 m using a numerical and deep learning algorithm. It is worth mentioning that the increase in *SINR_th_* requires a decrease in the distance between RFID and BS to achieve reliable data, which is a crucial issue for tracking infected people and helps decrease the probability of increasing COVID-19 infection cases.

From Figure 8, it can be observed that, for both the numerical and deep learning models, increasing the required *SINR_th_* decreases the required distance between an RFID reader and a BS to receive reliable and efficient information. For example, when the interference distance between an RFID and any transmitting devices (*d_IBS_*) is 50 m and *SINR_th_* is 6 dB, then the required distance between an RFID reader and a BS should be in the range of 8.95 m. However, the required distance between an RFID reader and a BS is 4.49 m if *SINR_th_* is 18 dB. Additionally, it can be mentioned that if the interference distances between an RFID reader and any transmitting devices decreases, the required distance between an RFID and a BS must decrease. For example, if *d_IBS_* is 50 m or 250 m and *SINR_th_* is 8 dB, then the required distance between an RFID reader and a BS is in the range of 7.79 m or 81.56 m, respectively. On the other hand, if *SINR_th_* is 20 dB, the required distance between an RFID reader and a BS will be approximately 11.1 m if *d_IBS_* is 100 m and is 4.49 m when *d_IBS_* 40.08 m. This is caused by the fact that increasing the interference distance leads to increasing the unreliability of receiving message due to receiving unwanted information. Under these circumstances, it is important to adapt the RFID distance with the position of BS, as during this pandemic and to allow feasible and accurate tracking the system should receive accurate data. 

It can be seen from the result presented in Figure 9, that increasing the required system QoS decreases the required transmission distance between an RFID reader and a BS. Additionally, it can be noticed that increasing the QoS with *SINR_th_* increasing dramatically decreases the required distance between an RFID reader and a BS, as this is considered as a very high network requirement that should be obtained with the minimum required distance to overcome the loss that could happen due to a long transmission distance. As it can be mentioned from Figure 9, for the five assumed *SINR_th_* which are 0, 5, 10, 15, and 20 dB if the required QoS is 0.9, the required transmission distance between an RFID reader and a BS will be approximately 11.13 m, 14.29 m, 19.09 m, 25.31 m and 34.47, respectively, using numerical and using the deep learning model. This result is correlated with the results obtained previously, which showed that to achieve a high system performance, the distance between an RFID reader and a BS should be adapted based on the system requirement and channel conditions.

Figure 10 describes how RFID transmission power affects the required distance between an RFID reader and a BS to receive reliable data about rquired COVID-19 information. In this scenario, the network parameters are assumed as follows: *d_IBS_* is 110 m and *SINR_th_* is 20 dB and there are two different QoS requirements. As can be depicted from Figure 10, increasing the RFID transmission power results in an increase in the required transmission distance between an RFID reader and BS, as increasing the transmission power should overcome the effect of other different parameters that may affect the transmission reliability such as interference and path loss. Under this circumstance, it is important to assure that the data received by the BS is sent by the maximum allowed power for the RFID, as this could affect the quality of the received information. 

As can be observed from Figure 11, increasing the RFID transmission power increases the overall system data rate for the numerical and the deep learning model results. This figure ascertains the result obtained in Figure 9; to achieve reliable and efficient COVID-19 information, the RFID reader applied for this mission should have a maximum transmission power. 

It can be concluded from Figure 12 and from the results obtained from the numerical and deep learning model that, to achieve the highest system QoS, the interference distance should be predicted to allocate the RFID reader at a suitable distance from the BS. This distance must be determined based on the RFID reader transmission power. For example, assuming the worst-case scenario, which is having an interference distance (*d_IBS_*) of 50 m and the required QoS of 0.999, then the required distance between an RFID reader and a BS should be less than 1 m when the transmission power (*P_R_*) is 0 dBm, while it is required to be 1.16 m when *P_R_* is 33 dBm. On the other hand, when the interference distance increases for example becomes 250 m, then the required distance between an RFID reader and a BS is 1.91 m for *P_R_* equals 0 dBm and between 12.42–13.11 m for *P_R_* equals 33 dBm.

Figure 13 shows how the estimated required transmission distance between an RFID reader and a BS enhances the overall system data rate. Additionally, it can be noticed, based on the assumed interference distance and *SINR_th_*, that increasing the required distance between an RFID reader and a BS increases the overall system data rate. This result reflects the effectiveness of the proposed approach based on the prediction of the required distance between an RFID reader and a BS increases the amount of received data per second. This is an important issue that should be taken into consideration, as increasing the amount of receiving data per second shows that the system overcomes any harmful received signal due to the channel conditions.

It can be concluded from the results obtained in Figure 6, Figure 7, Figure 8, Figure 9, Figure 10, Figure 11, Figure 12 and Figure 13 that adapting the required distance between an RFID reader and a BS for the given transmission conditions, such as interference distance (*d_IBS_*), channel quality in terms of *α* and an RFID reader transmission power (*P_R_*), enhances the system performance and increases the effectiveness and accuracy of the received COVID-19 information. The proposed approach provides effective guidance for deciding when and how the RFID reader should communicate with the BS to send COVID-19 information and whether this information is going to be stored or not. Therefore, based on the presented results, the enhancement of the communication performance between an RFID reader and a BS can be achieved by adaptively indicating the appropriate transmission distance between an RFID reader and a BS under different network and channel conditions.

Despite the good results obtained from the proposed approach, there is still some limitation that should be addressed. One of the important issues that should be addressed is the power limitation of WSN and the difficulties of recharging or changing batteries. Additionally, network failure or system overhead could happen when the amount of sent data increases. 

## 5. Conclusions

A reliable, efficient, accurate, and secured data transmission system was proposed for COVID-19 cases’ prediction and tracking using analytical and deep learning techniques. First, the proposed tracking system was described and explained based on different conditions. Then, the optimum required distance between an RFID reader and a BS, where the data should be handled, is calculated using the Lagrange optimization technique and simulated using MATLAB. A proposed deep learning model is trained using the generated simulations and is compared by way of several 10-fold cross-validation experiments with several benchmarks. Next, the analytics of the results of the required position of the RFID reader with respect to the BS to achieve reliable, efficient, and accurate data. It has been shown from the obtained results from analytical and deep learning that the proposed approach can exhibit the best performance under the different channel and environmental conditions. The effect of using the probability conditions on the number of transmitted record for storage has been explained and showed in graph. The problem of receiving accurate and reliable data by adapting the distance between an RFID reader and a BS under different parameters was discussed and solved using the Lagrange optimization technique and deep learning. Additionally, the effect of having different required *SINR_th_* and RFID transmission power on adapting the distance between an RFID reader and a BS was investigated. It has been proven that increasing the required *SINR_th_* leads to decreasing the required distance between an RFID reader and a BS. Moreover, it has been shown that increasing the RFID transmission power enhances the required system performance and increases the required distance between an RFID reader and a BS. Moreover, it has been proven that the proposed approach provides effective guidance for indicating how the communication between an RFID reader and a BS should be. Therefore, based on the presented results, indicating the appropriate transmission distance between an RFID reader and a BS leads to enhancing the required system performance and assuring that the received data are accurate, reliable, efficient, and secure to facilitate the COVID-tracking and detection.

## Figures and Tables

**Table 1:** Example of COVID-tracking actions based on the received data.

Conditions	Action
P(A) & P(B)	Received data should be stored in the database until it is confirmed that the target people are safe
P(A) & P(C)	Immediate Isolation and tracking of all people who were in close contact
P(A) & P(D)	Immediate Isolation and tracking of all people who were in close contact
P(A) & P(B) & P(C)	Immediate Isolation and tracking of all people who were in close contact
P(A) & P(B) & P(D)	Immediate Isolation and tracking of all people who were in close contact
P(A) & P(B) & P(C) & P(D)	Immediate Isolation and tracking of all people who were in close contact
P(E) & P(B)	There is no need to save the data
P(E) & P(C)	For safety, save the data until it is confirmed that the target people are safe
P(E) & P(D)	For safety, save the data until it is confirmed that the target people are safe
P(E) & P(B) & P(C)	For safety, save the data until it is confirmed that the target people are safe
P(E) & P(B) & P(D)	For safety, save the data until it is confirmed that the target people are safe
P(E) & P(B) & P(C) & P(D)	For safety, save the data until it is confirmed that the target people are safe
P(E) & P(F)	There is no need to save the data
P(E) & P(D)	There is no need to save the data
P(E) & P(D)	There is no need to save the data
P(E) & P(F) & P(C)	There is no need to save the data
P(E) & P(F) & P(D)	There is no need to save the data
P(E) & P(F) & P(C) & P(D)	There is no need to save the data

**Table 2:** System parameters.

Parameter	Value
*P_R_*	33 dBm [27]
*P_T_*	−21 dBm [27,40]
*B*	10 KHz [41]
*f*	915 MHz [27]
*G_tag_ G_reader_*	0.8 [27]
*P_I_*	23 dBm [38]
*γ_th_* and *β_th_*	20 dB
τ	0.8 [27]

**Table 3:** The statistical description of the dataset.

	U	$P_{R}$	*SINR_th_*	$d_{IBS}$	$d_{RBS}$
Number of records	90,288	90,288	90,288	90,288	90,288
Mean	0.920	23.698	15.471	122.831	12.862
Standard Deviation	0.158	11.010	6.433	68.597	17.061
Minimum	0.100	0.000	0.000	1.000	0.001
Maximum	0.999	33.000	20.000	250.000	129.721

**Table 4:** Results of average 10-fold cross-validation for the benchmarks and the proposed model.

	MAE		MSE	
	Train	Test	Train	Test
LR	6.05	6.05	81.67	81.68
Ada	5.03	5.04	42.1	42.36
SVR	1.16	1.16	7.74	7.75
MLP	1.09	1.09	3.71	3.73
Proposed model	0.18	0.18	0.09	0.09

## Data Availability

Not applicable.
